# Running into differences

**DOI:** 10.7554/eLife.101013

**Published:** 2024-07-31

**Authors:** Eleni Psarou, Shivangi Patel, Marieke Schölvinck

**Affiliations:** 1 Ernst Strüngmann Institute for Neuroscience Frankfurt Germany

**Keywords:** marmoset, visual cortex, locomotion, electrophysiology, vision, Mouse, Rhesus macaque, Other

## Abstract

Body movement does not significantly increase neuronal activity in the primary visual cortex of marmosets, in contrast to the effects observed in mice.

**Related research article** Liska JP, Rowley DP, Nguyen TTK, Muthmann JO, Butts DA, Yates JL, Huk AC. 2023. Running modulates primate and rodent visual cortex differently. *eLife* **12**:RP87736. doi: 10.7554/eLife.87736.

Imagine you are walking down a busy street. As you move, your brain receives visual information that it uses, alongside other sensory inputs, to guide your next steps. Traditionally, it was thought that the parts of the brain that process sensory information, such as the primary visual cortex, were largely unaffected by body movements. It therefore came as a shock when a group of researchers discovered that running significantly increased neuronal activity in the primary visual cortex of mice (Niell and Stryker, 2010; Figure 1A).

**Figure 1.**
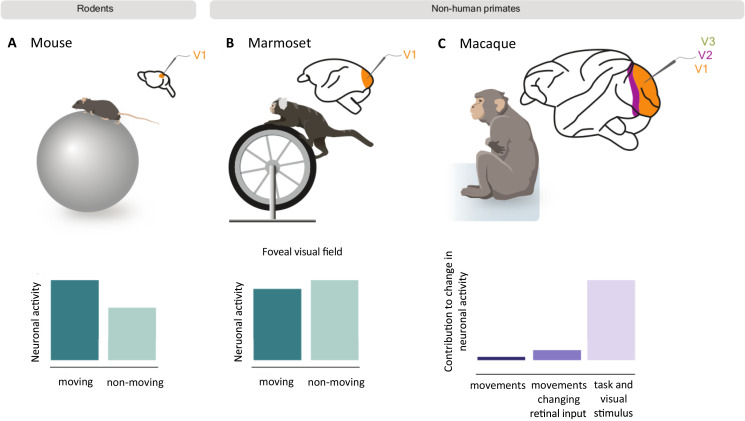
How movement impacts neuronal activity in the visual cortex of rodents and non-human primates. Mice (**A**) and marmosets (**B**) were placed on a rotating treadmill (top) with their heads fixed in position so they could view visual stimuli on a screen. The neuronal activity of their primary visual cortex was then recorded as they ran or sat still. In mice, neuronal activity was higher when running (dark green bar) compared to when the mice were still (light green bar; bottom left graph). However, movement did not cause the same effect in marmosets, and slightly decreased the activity of neurons that respond to the foveal visual field (bottom middle graph). A similar study in macaques (**C**) also found that running only slightly increased neuronal activity in the primary (V1) and mid-level (V2 and V3) visual cortex. The bar graph shows how much of this change in activity was the result of spontaneous body movements (dark purple), movements that change visual inputs to the retina (purple), and visual stimuli and task-related variables (light purple). Panel A and B are schematic representations of the results from Liska et al., 2023; panel C represents the results from Talluri et al., 2023.

This rise in activity was different from the increase caused by arousal (Vinck et al., 2015), suggesting that the observed change in the primary visual cortex was due to the motion of the body. However, it remained unclear whether body movement also influences sensory processing in non-human primates, which are evolutionarily closer to humans. Now, in eLife, Jacob Yates, Alexander Huk and co-workers – including John Liska and Declan Rowley as joint first authors – report how running impacts the primary visual cortex of marmosets (Liska et al., 2023).

The team (who are based at the University of Texas at Austin, University of Maryland, University of Berkeley, California, and UCLA) recorded the neuronal activity of marmosets as they ran or remained stationary on a treadmill whilst looking at a screen, similar to the experimental set-up used in the previous mouse study (Figure 1B). The results were then compared to publicly available datasets from the experiments conducted in mice.

Analysis of the mouse dataset confirmed that body movements increase neuronal activity in the primary visual cortex, in agreement with previous studies. However, Liska, Rowley et al. found that running did not cause the same pronounced effect in marmosets. Intriguingly, the behavior of neurons in the primary visual cortex depended on which parts of the visual field they respond to: movement decreased the activity of foveal neurons, which receive inputs from the center of the visual field, but slightly increased the activity of peripheral neurons, which respond to inputs from the outermost parts of the visual field. Unlike humans and non-human primates, rodents do not have a fovea, which may contribute to the observed difference between the marmosets and mice.

Another possibility is that rodents and monkeys process non-visual inputs (such as running or attention) differently in their visual cortices. To test this, Liska, Rowley et al. applied a model to their data which tests to what degree non-visual inputs adjust the way neurons respond to visual stimuli. The team found that the model was able to explain a significant proportion of the data from both the marmosets and mice, including the variations they had observed between experimental trials in each species. This suggests that the different response to body movement between the marmosets and mice is not because they process non-visual factors in distinct ways.

A recent study similarly showed that body movement only minimally increases neuronal activity in the visual cortex of macaques, another non-human primate (Talluri et al., 2023; Figure 1C). The researchers found that only a small fraction of the activity in the visual cortex was due to spontaneous body movements (such as scratching). Instead, the majority was explained by movements which changed the visual inputs arriving to the brain, or visual stimuli and task-related variables, such as when the monkey received a reward.

So, how do such striking differences between mice and two primate species come about? Liska, Rowley et al. propose several possible mechanisms. First, the distinction may be due to neurons in the primary visual cortex responding differently to the neurotransmitter acetylcholine: in primates, acetylcholine generally suppresses activity in the visual cortex, but in rodents, it increases activity (Disney et al., 2007; Pakan et al., 2016). Second, because the visual system of a primate has more specialized areas than the rodent visual system, factors like running may influence those regions rather than condensing its effects to the primary visual cortex. Third, the neurons in the primary visual cortex of mice may be more sensitive to running because they receive substantial direct projections from premotor areas of the brain which plan and organize movement (Leinweber et al., 2017) – this is note the case for the neurons in the primary visual cortex of monkeys (Markov et al., 2014).

The findings of Liska, Rowley et al. open up several exciting avenues for exploring how the brain integrates sensory inputs with movements. Categorizing the movements as relevant or irrelevant to the task at hand might shed light on which aspects of non-visual inputs affect sensory areas the most. More generally, by investigating how visual areas in mice and monkeys respond to non-visual inputs, it may be possible to uncover overarching mechanisms that are used by sensory systems to integrate and sample information from the environment.
